# Prospective, Randomized Ex Vivo Trial to Assess the Ideal Stapling Site for Endoscopic Fundoplication with Medigus Ultrasonic Surgical Endostapler

**DOI:** 10.1155/2016/3161738

**Published:** 2016-07-31

**Authors:** Tae-Geun Gweon, Kai Matthes

**Affiliations:** ^1^Division of Gastroenterology, Children's Hospital Boston, Harvard Medical School, Boston, MA 02115, USA; ^2^Department of Anesthesiology, Perioperative and Pain Medicine, Boston Children's Hospital, Harvard Medical School, Boston, MA 02115, USA

## Abstract

*Background and Aims*. Endoscopic fundoplication is an emerging technique for the treatment of gastroesophageal reflux disease (GERD). The aim of this study is to determine the ideal position of the staples in relation to gastroesophageal junction (GEJ).* Methods*. Ten endoscopic fundoplication procedures were performed in each group using fresh ex vivo porcine stomachs: Group A: 2 staples each at 3 cm above the GEJ and 180° apart; Group B: 2 staples at 3 cm and 90° apart; Group C: 2 staples at 4 cm and 180° apart; Group D: 3 staples at 3 cm with 90° between each staple (180° total). After the procedure, the stomach was gradually filled with water. Gastric yield pressure (GYP) was determined by detection of reflux of the water in esophagus or by rupture of staples.* Results*. Mean increase of GYPs (±SD) after the procedure was as follows: Group A: 16.9 ± 8.7; Group B: 8.1 ± 7.9; Group C: 12.2 ± 9.4; Group D: 22.7 ± 13.3. GYP in Group A and Group D was higher than Group B (*p* = 0.03 and *p* = 0.01, resp.).* Conclusions*. We recommend the placement of 3 staples at 3 cm distance from the GEJ, which resulted in the highest increase of GYP.

## 1. Introduction

Gastroesophageal reflux disease (GERD) is a problematic condition, which develops when reflux of gastric contents causes clinical symptoms and complications [1]. Proton pump inhibitors (PPIs) are the first-line therapy in the treatment of GERD. However, unresponsiveness of PPI is known to be at 20% [2]. GERD is considered a chronic and relapsing disease. Patients with GERD suffer symptoms after discontinuation of medication [3]. Therefore, patients with GERD usually require long-term treatment with PPIs including both of maintenance therapy and on demand therapy. However, long-term therapy with PPIs is often associated with poor patient compliance and has potential chance of adverse events [4–6]. Long-term prescription of PPIs is also a burden to health-care system [7]. Therefore, it is prudent to develop an alternative therapy for GERD to avoid long-term PPI medication with clinical improvement of symptoms without PPI therapy.

Recently, the Medigus Ultrasonic Surgical Endostapler (MUSE*™*; Medigus, Omer, Israel), a combined video- and ultrasound-guided transoral surgical stapler, has been cleared by the US Food and Drug Administration and CE marked for use in the European Union for endoscopic fundoplication for the treatment of GERD in patients who require and respond to PPI. Patients with any of following conditions are contraindicated for MUSE: (1) hiatal hernia > 3 cm, (2) failure to reduce hernia with positive end-expiratory pressure up to 10 cm H_2_O, (3) stricture or varices in the esophagus, (4) body mass index (kg/m^2^) < 21 or > 35, and (5) nonresponders to PPI therapy. Several studies showed favorable short-term and long-term efficacy of endoscopic fundoplication using MUSE [8, 9]. However, there is no guideline for ideal stapling location to date, beyond recommendations in the device instructions for use to place the staples between 2.5 and 3.5 cm above the gastroesophageal junction (GEJ).

The EASIE-R simulator (Endosim LLC, Hudson, Mass) is a validated bench model using ex vivo porcine stomachs, which has been used effectively for testing various medical devices [10–12]. The advantage of a bench model is that in a simulated environment a procedure can be repeated in a controlled standardized setting to focus on the technical functionality of a procedure. The aim of this study was to determine the ideal stapling position in relation to the GEJ in this validated simulation model.

## 2. Materials and Methods

### 2.1. Endoscopic Fundoplication with MUSE

Endoscopic fundoplication can be performed with the MUSE. The MUSE system consists of an endostapler, staple cartridge, monitor, and console unit. The distal tip of MUSE contains an anvil for stapling, a miniature video camera, and an ultrasound transducer. The insertion length of the MUSE is indicated on the shaft of the device. After the staple cartridge is loaded, the operator types the insertion length at the level of the GEJ into the console unit. Then, the monitor shows the location of the stapling position, which is programmed to be at 3 cm above GEJ. The distal shaft of the MUSE can be retroflexed to 270°, which lifts the proximal part of fundus and enables the stapling of stomach to distal esophagus. The MUSE is disposable, but stapling can be performed several times by replacing the staple cartridges during the case. Each time the stapler is fired, also called “stapling,” it delivers five closely spaced staples.

### 2.2. Study Design and Procedure

**Figure 1:**
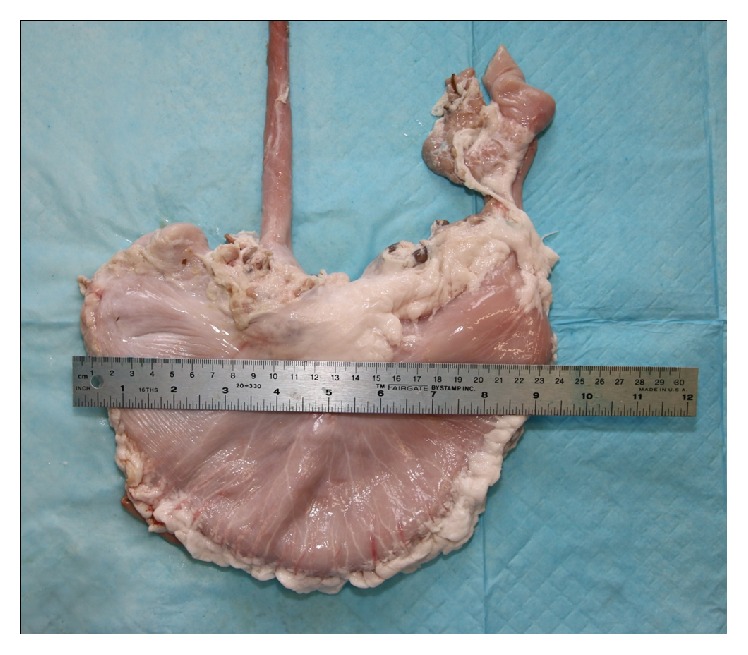
Measurement of horizontal length of stomach.

This prospective randomized experimental study has been exempted from institutional review because no human or animal subjects were involved. The inanimate models were obtained from a commercial food supplier. Four different stapling techniques were compared by varying the distance of stapling location from the GEJ and the angle between the staples in the horizontal plane: Group A: 2 staples each at 3 cm distance, angle 180 degrees; Group B: 2 staples at 3 cm, angle 90 degrees; Group C: 2 staples at 4 cm, angle 180 degrees; Group D: 3 staples at 3 cm, 90 degrees between each staple quintuplet (180 degrees total). Randomization was done by a computer-generated randomization list. A paper, which disclosed the procedure group, was sealed in an opaque envelope. Before the procedure, horizontal length of the stomach was measured (Figure 1). After ex vivo stomach was placed in the EASIE-R simulator, envelope was opened. The start time of procedure was determined when GEJ was identified by the MUSE. The end time was the point when the MUSE was pulled out from the esophagus. The procedure was done by one experienced endoscopist with more than 4 years of experience and one assistant. Before the trial, the operator performed 5 cases of endoscopic fundoplication for practice.

### 2.3. Efficacy of the Endoscopic Fundoplication

Efficacy of the procedure was assessed by gastric yield pressure (GYP) and gastric yield volume (GYV) [13, 14]. To measure GYP and GYV, 18-gauge cannula was inserted into stomach lumen, which was connected to a pressure transducer (Propaq® Encore; Welch Allyn, NY, USA). The pylorus was tightly closed around the tubing of the roller pump and the stomach was gradually filled with methylene-dyed normal saline using a roller pump with 600 mL/min. The GYP was defined as intragastric pressure when reflux of the methylene-blue dyed water was detected in the esophagus with a gastroscope positioned 3 cm above stapling site. If the pressure led to a rupture of the specimen, this burst pressure threshold was noted as GYP. The GYV was defined as total amount of infused water to the point of reflux detection (GYP point). GYP and GYV were measured before and after the procedure. All documentation of GYP and GVV including procedure time was recorded by the assistant.

### 2.4. Statistical Analysis

Statistical analyses were conducted using SPSS 18.0 software (SPSS Inc., Chicago, Ill). Continuous variables were documented as mean and standard deviation or interquartile range (IQR). Increase of GYP was compared between the four groups. For this statistical analysis, we used the repeated measures ANOVA. GYV between before and after the procedure were compared in each group using Wilcoxon signed-rank test. Categorical variables were compared using Chi-square test and Fisher exact test. A *p* value < 0.05 was considered statistically significant.

## 3. Results

**Figure 2:**
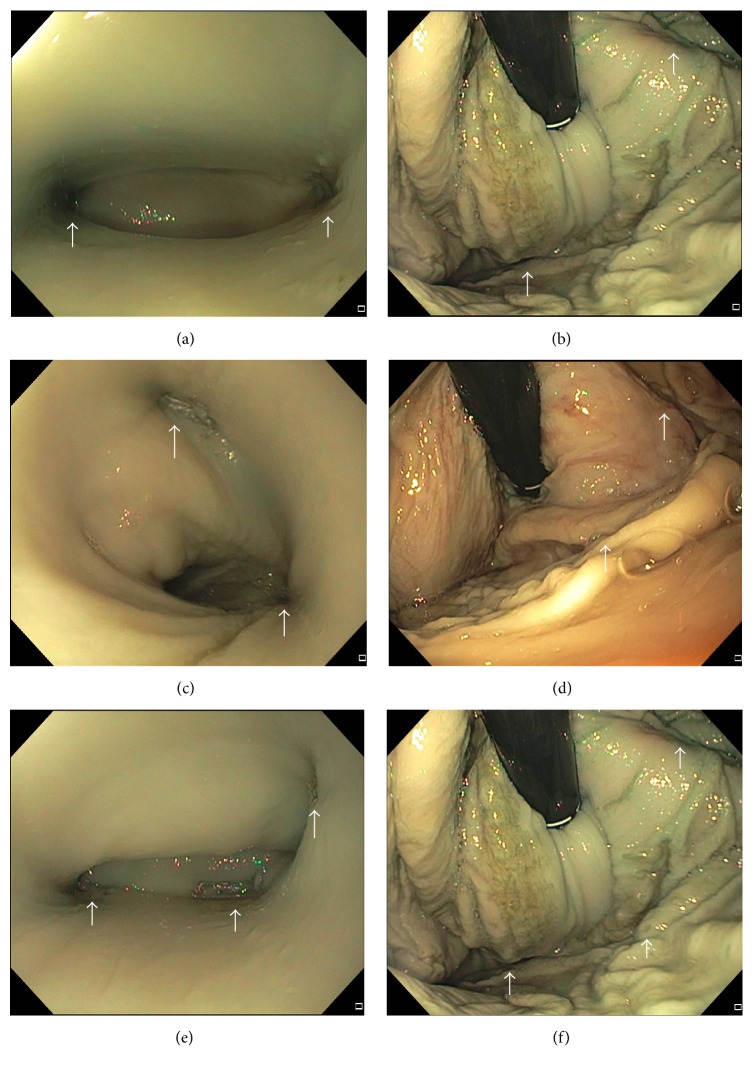
Endoscopic imaging following endoscopic fundoplication. White arrows indicate stapling site. (a) 2 staples with 180° at 3 cm distance from the GEJ, esophageal view. (b) 2 staples with 180° at 3 cm distance from gastroesophageal junction, retroflexed view. (c) 2 staples with 90° at 3 cm distance from gastroesophageal junction, esophageal view. (d) 2 staples with 90° at 3 cm distance from gastroesophageal junction, retroflexed view. (e) 3 staples with 180° total at 3 cm distance from gastroesophageal junction, esophageal view. (f) 3 staples with 180° total at 3 cm distance from gastroesophageal junction, retroflexed view.

A total of 40 endoscopic fundoplication procedures, 10 procedures per each group, were successfully performed. The shape of the stomachs used in the study were consistent with no difference in average specimen diameter or length between each group. The length of the specimen (axis from GEJ to pylorus) in each group was as follows: Group A: 25.0 ± 1.9 cm; Group B: 26.0 ± 3.2 cm; Group C: 25.5 ± 2.4 cm; Group D: 26.0 ± 3.1 cm. A Hill grade I valve, defined as the close approximation of the cardia to the shaft of the endoscope, was created after the endoscopic fundoplication procedures in all specimens (Figure 2). The procedure time in each group was as follows: Group A: 23.8 ± 7.6 min; Group B: 24.2 ± 10.1 min; Group C: 20.2 ± 6.2 min; Group D: 33.2 ± 9.7 min. The procedure time in Group D was longer than Group A and Group C (*p* = 0.03, *p* < 0.01, resp.).

### 3.1. Gastric Yield Pressure

Baseline GYP before the procedure was 0 mmHg in all groups. GYP after the procedure was as follows: Group A: 16.9 mmHg (IQR, 10.8–22.3); Group B: 8.1 mmHg (IQR, 1.8–14.3); Group C: 12.2 mmHg (IQR, 3.3–20.0); Group D: 22.7 mmHg (IQR, 11.5–35.0). Pressure increase of Group A and Group D was higher than Group B (Group A versus Group B, *p* = 0.03; Group B versus Group D, *p* = 0.01). Incidents of burst pressure being lower than GYP occurred in 6 cases, 4 cases, 5 cases, and 7 cases of each group, respectively. Total rupture rate was 55%. The rupture rate did not differ between all groups.

### 3.2. Gastric Yield Volume

Mean GYV before the procedure was as follows: Group A: 274.0 mL (IQR, 177.5–325.0); Group B: 272.0 mL (IQR 172.5–327.5); Group C: 319.0 mL (IQR, 207.5–420.0); Group D: 316.0 mL (IQR, 207.5–420.0). Mean GYV after the procedure is as follows: Group A: 2299.0 mL (IQR, 1497.5–3127.5); Group B: 1663.0 mL (IQR 862.5–2565.0); Group C: 1857.0 mL (IQR, 1175.0–2865.0); Group D: 2639.0 mL (IQR, 1907.5–3200.0). In all groups, the GYV was significantly larger than before the procedure (*p* < 0.01).

## 4. Discussion

GERD is chronic, not curable, and easily relapses after discontinuation of drugs [15, 16]. Laparoscopic fundoplication is one of the most common alternative therapies for GERD and has been investigated in various clinical trials to demonstrate favorable efficacy and reduce adverse events [17, 18]. Laparoscopic fundoplication showed similar long-term efficacy compared to PPI [19]. Magnetic sphincter augmentation, electrical stimulation of lower esophageal sphicter (LES), and endoscopic fundoplication are emerging treatment for GERD [9, 20, 21]. However, long-term follow-up studies are rare to date on these treatment options above.

Endoscopic treatment of GERD included suturing of proximal stomach [14], endoscopic submucosal dissection [22], and endoscopic fundoplication [9, 13, 23]. Of these, endoscopic fundoplication showed favorable short-term efficacy, but long-term efficacy was not proved [9, 24]. Recently, devices for endoscopic fundoplication by stapling the distal esophagus and cardia of stomach have become available. Compared to surgical treatment, endoscopic treatment has advantage of less invasiveness. Use of endoscopic fundoplication is expected to increase. To obtain better long-term efficacy of the endoscopic fundoplication, it is important to determine an ideal stapling position.

In the underlying study, we compared various stapling position to determine ideal stapling position for the endoscopic fundoplication using MUSE. Our results showed that three staples showed the highest increase of GYP among all groups. Two staples placed 3 cm above GEJ with 180° showed a higher increase of GYP in comparison to 2 staples placed 3 cm above the GEJ with 90° between each staple. This is the first study investigating the ideal stapling position for endoscopic fundoplication.

In a preliminary study, we placed staples 2 cm above the GEJ which resulted in an insufficient increase of GYP; therefore we did not investigate a distance of 2 cm or less in this study [25]. We found that the endoscopic fundoplication created a sufficient valve at the GEJ by lifting the cardia of the stomach and stapling it to distal esophagus. Even though all four groups resulted in a Hill grade I valve, we still found significant differences in GYP among the tested four groups. The use of three staples in comparison to two staples showed the largest increase of GYP. Among 2 plications, placing the staples at a distance of 3 cm from the GEJ with a 180° between the staples appeared to be the most effective. We found an efficient valve with the 180° staple position which appeared tighter than the stapling with 90°. Among the stapling group with 180°, the 3 cm distance to the GEJ group showed a better result than the 4 cm distance group. This outlines that the distance between GEJ and stapling site is very important to assure the highest increase in GYP. A too long distance of stapling location from the GEJ created a too loose valve. Our results demonstrate that the most efficient distance to the GEJ is at 3 cm. In about 60% of cases a rupture of the ex vivo specimen occurred during the inflation of the stomachs with water. The rupture rate was similar among all groups. This phenomenon would unlikely be seen in an actual clinical situation because the extent of dilation observed in the ex vivo study is more than physiological since we infused more than 2 liters. To date, a rupture at the stapling site during or following a endoscopic fundoplication has not been reported [8, 9]. We suggest that the staples have a favorable mechanical strength to prevent reflux and unlikely result in a rupture of the stomach wall at the staple site.

Some limitations of this study have to be addressed. First, we have used ex vivo models in this study. Postprocedural clinical symptoms such as dysphagia, intra- or postoperative bleeding, and perforation are important potential adverse events, but the detection of these were not possible in this study. We were able to investigate technical feasibility of the procedure. Adverse events should be investigated in a clinical trial. Second, all the procedures were performed by one endoscopist and one assistant. However, we believe that interoperator variation would be minimal in using this technique. Third, intrinsic LES pressure was not investigated. In the previous studies, patient's symptom was improved although LES pressure was not increased significantly after the endoscopic fundoplication [9, 26]. Even though LES incompetency is important in the pathogenesis of GERD, which is multifactorial, further study is needed to assess the influence of the intrinsic pressure on LES function in the setting of GERD.

In conclusion, we were able to demonstrate significant differences in GYP in comparison to stapling position with the highest increase in GYP observed with the placement of 3 staples at 3 cm distance from the GEJ with an angle of 90 degrees between each staple position (1st and 2nd; 2nd and 3rd).
